# Clinical relevance of serum lipids in the carcinogenesis of oral squamous cell carcinoma

**DOI:** 10.1186/s12903-023-02859-6

**Published:** 2023-04-03

**Authors:** Ling Qian, Bo Qian, Juanyong Xu, Jingjing Yang, Guoying Wu, Yuping Zhao, Qinglan Liu, Zhiran Yuan, Yuan Fan, Huaiqi Li

**Affiliations:** 1 Department of Oral Mucosal Diseases, The Affiliated Stomatological Hospital of Nanjing Medical University, Nanjing, China; 2 Jiangsu Province Key Laboratory of Oral Diseases, Nanjing Medical University, Nanjing, China; 3 Jiangsu Province Engineering Research Center of Stomatological Translational Medicine, Nanjing, China; 4 Department of Cardiothoracic Surgery, Children’s Hospital of Nanjing Medical University, Nanjing, China; 5 The Affiliated Stomatological Hospital of Nanjing Medical University, Nanjing, China; 6 Department of Oral and Maxillofacial Surgery, The Affiliated Stomatological Hospital of Nanjing Medical University, Nanjing, China

**Keywords:** Oral squamous cell carcinoma (OSCC), Carcinogenesis, Lipids, Forecasting

## Abstract

**Background:**

Dyslipidaemia is associated with cancers. However, the specific expression of serum lipids in oral potentially malignant disorders (OPMD) and oral squamous cell carcinoma (OSCC) remains unclear, and it remains unknown whether serum lipids are associated with the development of OPMD and OSCC. This study investigated the serum lipid profiles of OPMD and OSCC patients, and the association of serum lipids with the occurrence of OPMD and OSCC.

**Methods:**

A total of 532 patients were recruited from the Affiliated Hospital of Stomatology, Nanjing Medical University. Serum lipid parameters including total cholesterol (TC), triglycerides (TGs), high-density lipoprotein cholesterol (HDL-C), low-density lipoprotein cholesterol (LDL-C), apolipoprotein A (Apo-A), apolipoprotein B (Apo-B), and lipoprotein (a) (Lpa) were analysed, and clinicopathological data were collected for further analysis. Furthermore, a regression model was used to evaluate the relationship between serum lipids and the occurrence of OSCC and OPMD.

**Results:**

After adjusting for age and sex, no significant differences were observed in serum lipid or body mass index (BMI) between OSCC patients and controls (*P* > 0.05). HDL-C, Apo-A, and Apo-B levels were lower in OSCC patients than in OPMD patients (*P* < 0.05); HDL-C and Apo-A levels were higher in OPMD patients than in controls (*P* < 0.05). Furthermore, female OSCC patients had higher Apo-A and BMI values than males. The HDL-C level was lower in patients under 60 years of age than in elders (*P* < 0.05); and age was related to a higher risk of developing OSCC. Female patients with OPMD had higher TC, HDL-C, and Apo-A levels than males (*P* < 0.05); OPMD patients over 60 years of age had higher HDL-C than youngers (*P* < 0.05), whereas the LDL-C level was lower in elders (*P* < 0.05). The HDL-C and BMI values of the patients with oral leukoplakia (OLK) with dysplasia were more elevated than those of the oral lichen planus group, and the LDL-C, and Apo-A levels in patients with OLK with dysplasia were decreased (*P* < 0.05). Sex, high HDL-C and Apo-A values were associated with the development of OPMD.

**Conclusion:**

Serum lipids exhibited certain differences according to the occurrence and development of OSCC; high levels of HDL-C and Apo-A might be markers for predicting OPMD.

## Background

Oral squamous cell carcinoma (OSCC) is a common malignant subtype of oral cancer which had an incidence of approximately 377 000 cases worldwide according to GLOBOCAN 2020 [1]. Despite continuous advances such as radiotherapy plus cetuximab, and the treatment of anti–programmed death 1 monoclonal anti-body in head and neck squamous cell carcinoma (HNSCC) patients in the clinical management, the patients with HNSCC have a poor survival [2–4]. Moreover, oral potentially malignant disorders (OPMD), which are a recognizable type of clinically suspicious lesion that has the risk of progressing to cancer, are a group of diseases that may precede the development of OSCC [5]. OPMD mainly include oral leukoplakia (OLK), oral lichen planus (OLP), erythroplakia, erythroleukoplakia, proliferative verrucous leukoplakia (PVL), oral submucous fibrosis (OSF), and lupus erythematosus [6, 7]. According to the known literature, the overall malignant transformation rate across all OPMD is 7.9% [8]. Therefore, it is important to investigate the process of transformation of OPMD to OSCC to improve the diagnosis and treatment.

Metabolic reprogramming is a common characteristic of metastasis and progression in cancers [9, 10]. The high rate of de novo synthesis in lipid metabolism guarantees that cancer cells sustain rapid growth and faster proliferation [11–13]. Investigators found that serum total cholesterol (TC) levels had an impact on liver tumour development in mice [14]. Dyslipidaemia increased the risk in non-small cell lung cancer patients [15, 16]. Triglycerides (TGs) and TC might be potential predictors of overall survival for cervical cancer patients [17]. There is evidence that cholesterol and its intermediates are upregulated in cancer due to the activation of oncogenic signalling pathways, such as the prevention of apoptosis, ROS generation, and the promotion of angiogenesis. Some studies suggest that low-density lipoprotein cholesterol (LDL-C) is positively correlated with the risk of cancer development, while high-density lipoprotein cholesterol (HDL-C) has a negative effect on cancer [18]. Apolipoprotein A (Apo-A) was associated with a lower risk of hepatic flexure cancer [19]. Another study indicated that high levels of apolipoprotein B (Apo-B) were associated with a twofold increase in the risk for recurrence, and there was no relationship between Apo-A and breast cancer outcomes [20]. Moreover, Li et al. found that TC, LDL-C, Apo-A and Apo-B levels were significantly lower in patients with HNSCC than in matched control individuals, and high lipoprotein (a) (Lpa) was associated with a worse prognosis [21]. However, another study found that HDL-C was the only lipoprotein that showed significant differences between healthy control individuals and patients with HNSCC, and that high LDL-C levels favoured the survival of HNSCC patients [22]. Gupta et al. found that there was a decrease in TC, HDL-C, and TG in the patients with the precancerous lesions and OSCC as compared to the controls [23]. Another study also indicated that 20 OSF patients and 20 OSCC patients had lower levels of TC, HDL-C and LDL-C than normal subjects [24], but the sample size of the OPMD and OSCC were small. Thus, dyslipidaemia might occur in HNSCC and OPMD patients, but the expression profile of dyslipidaemia could not be clarified.

This study analysed blood samples from patients with OSCC, OPMD and controls. The serum lipid profiles were mapped and the associations between BMI and clinicopathological features were investigated to identify changes among the patients with OSCC and OPMD and controls. Furthermore, this study provided new insights and a basis for the clinical surveillance of the occurrence and development of OSCC.

## Methods

### Patient samples

A total of 187 OSCC patients, 200 patients in the control group with benign lesions (cysts, maxillofacial trauma, and impacted teeth) (control group), and 145 OPMD patients (105 OLP patients; 40 OLK with dysplasia patients) were selected between 2019 and 2021 from the Department of Oral and Maxillofacial Surgery or the Department of Oral Mucosal Diseases, Affiliated Hospital of Stomatology, Nanjing Medical University. The inclusion criteria were as follows: (1) OSCC patients needed to be definitively diagnosed by histopathology, and the OPMD patients or control group patients needed to be diagnosed clinically or pathologically; (2) OSCC patients were not treated with surgery, chemoradiotherapy, or targeted therapy, and patients with OPMD had not used relevant therapeutic agents; (3) all subjects had not taken any medications that could affect blood lipids for at least three months prior to blood sampling; and (4) liver, renal and thyroid function tests of all subjects were normal. The exclusion criteria were as follows: (1) patients who had diabetes, myxoedema, stroke, adrenal hyperfunction, coronary heart disease, polycystic ovary syndrome and diseases associated with abnormal lipid metabolism; (2) patients who were diagnosed with other neoplasms; (3) patients who had familial hyperlipidaemia; (4) patients with incomplete information; and (5) patients who were pregnant.

For data analysis, data such as sex, age, body height, body weight, locations and types of affected diseases, diagnosis, tumour stage and the serum levels of TC, TG, HDL-C, LDL-C, Apo-A, Apo-B, and Lpa were recorded for all the participants. All subjects involved in this study had been informed about the study and provided written informed consent at the time of their visit. These patients agreed to participate in this study, and this study was approved by the ethics committee of the Affiliated Hospital of Stomatology, Nanjing Medical University (PJ2018-073-001).

### Data collection

Venous blood samples were obtained from all subjects in the morning after 12–14 h of fasting. The blood parameters were measured by a chemical analyser (BECKMAN-COULTER AU680) based on spectrophotometric principles, and quality control was conducted daily before initiating analyses. Laboratory reference values for serum lipids were as follows: TC: 0-5.2 mmol/L; TGs: 0.4–1.81 mmol/L; HDL-C: 1.07–1.9 mmol/L; LDL-C: 0-3.1 mmol/L; Apo-A: 1-1.6 g/L; Apo-B: 0.1–1.81 g/L; and Lpa: 0-300 mg/L.

Body mass index (BMI) was calculated using the formula BMI = weight (kg)/ height^2^ (m^2^) (normal range: 18.5 kg/m^2^-23.9 kg/m^2^).

### Statistical analysis

Pairwise comparison analyses among the three groups at each serum lipid each expression level and BMI, adjusted for age and sex, were carried out by using the Tukey method in R 4.0.5. A logistic regression model was also used to test whether dyslipidaemia increased the risk of developing OSCC or OPMD. Clinicopathological parameters of OSCC patients and OPMD patients were analysed by SPSS 25.0, and all data are presented as the mean ± standard deviation. Independent-sample t tests were used for two-group comparisons, and one-way ANOVA was performed for three-group comparisons. *P* < 0.05 was considered significantly different.

## Results

### The levels of serum lipids and BMI among the OSCC, OPMD and control groups

The clinical and serological findings of the three groups are shown in Table 1. Considering the effects of age and sex, we adjusted the two factors using statistical methods, and the Tukey method was performed on the serum lipids and BMI among the three groups. No significant differences were observed in serum lipids or BMI values between the OSCC and control groups. The levels of HDL-C and Apo-A in the OPMD group were significantly higher than those in the control group. Interestingly, the expression levels of HDL-C, Apo-A, and Apo-B were significantly different between OPMD and OSCC patients (*P* < 0.05) (Table 2).

**Table 1:** Clinical data about the three groups of all participants

	Control (n = 200)	OSCC (n = 187)	OPMD (n = 145)
Gender (n)			
Male	116	117	42
Female	84	70	103
Age	52.83 ± 11.52	61.33 ± 10.98	51.70 ± 13.89
TC (mmol/L)	4.43 ± 0.91	4.51 ± 0.94	4.68 ± 0.87
TG (mmol/L)	1.31 ± 0.71	1.40 ± 0.96	1.32 ± 0.67
HDL-C (mmol/L)	1.18 ± 0.28	1.19 ± 0.32	1.49 ± 0.59
LDL-C (mmol/L)	2.46 ± 0.71	2.55 ± 0.78	2.38 ± 0.75
Apo-A (g/L)	1.32 ± 0.23	1.28 ± 0.30	1.49 ± 0.22
Apo-B (g/L)	0.91 ± 0.28	0.88 ± 0.29	0.97 ± 0.38
Lpa (mg/L)	196.05 ± 232.33	202.75 ± 261.60	172.30 ± 244.95
BMI (kg/m^2^)	23.34 ± 3.08	23.12 ± 3.31	23.81 ± 3.36

**Table 2:** Expression of serum lipid components and BMI among three groups ^a^

	Control	OSCC	OPMD	*P1*	*P2*	*P3*
TC (mmol/L)	4.24 ± 2.69	4.31 ± 1.37	4.45 ± 1.20	0.766	0.104	0.400
TG (mmol/L)	1.46 ± 2.83	1.62 ± 1.37	1.50 ± 1.32	0.260	0.927	0.551
HDL-C (mmol/L)	0.85 ± 1.13	0.82 ± 0.55	1.12 ± 0.48	0.757	< 0.01*	< 0.01*
LDL-C (mmol/L)	2.64 ± 2.12	2.77 ± 1.09	2.56 ± 0.96	0.234	0.607	0.052
Apo-A (g/L)	1.27 ± 0.71	1.23 ± 0.41	1.40 ± 0.36	0.240	< 0.01*	< 0.01*
Apo-B (g/L)	0.91 ± 0.85	0.88 ± 0.41	0.98 ± 0.48	0.544	0.114	0.015*
Lpa (mg/L)	175.83 ± 720.54	178.71 ± 358.96	154.01 ± 333.67	0.993	0.711	0.682
BMI (kg/m^2^)	22.79 ± 9.48	22.48 ± 4.65	23.28 ± 4.33	0.641	0.363	0.097

^a^ Data were adjusted for gender and age level, analyzed by the Tukey method

P1: P value compared with OSCC patients and Control patients

P2: P value compared with OPMD patients and Control patients

P3: P value compared with OPMD patients and OSCC patients

^*^P < 0.05

### Comparison of serum lipids and BMI according to different clinicopathological parameters of OSCC

Female patients had higher Apo-A and BMI values than male patients in the OSCC group (*P* < 0.05). Moreover, the HDL-C levels were lower in patients who were under 60 years of age than in those who were over 60 years of age (*P* < 0.05). However, there were no significant differences in lipid levels or BMI values between patients with different tumour-node-metastasis (TNM) stages or between patients with or without lymph node metastasis (*P* > 0.05). The sites of cancer occurrence (tongue, buccal, lip, palate, mandible, and gingiva) were not associated with the levels of TC, TG, HDL-C, LDL-C, Apo-A, Apo-B, Lpa or BMI (*P* > 0.05) in OSCC patients (Table 3).

**Table 3:** Comparison of serum lipids and BMI between different subgroups in clinicopathologic characteristics of OSCC patients

Parameter(n)	TC (mmol/L)	TG (mmol/L)	HDL-C (mmol/L)	LDL-C (mmol/L)	Apo-A (g/L)	Apo-B (g/L)	Lpa (mg/L)	BMI (kg/m^2^)
Gender								
Male (117)	4.42 ± 0.98	1.48 ± 1.57	1.17 ± 0.36	2.49 ± 0.82	1.23 ± 0.31	0.86 ± 0.31	186.44 ± 224.46	22.70 ± 3.18
Female (70)	4.65 ± 0.86	1.42 ± 0.67	1.23 ± 0.25	2.66 ± 0.71	1.35 ± 0.25	0.91 ± 0.24	230.00 ± 313.95	23.83 ± 3.43
*P*	0.094	0.752	0.166	0.169	0.011*	0.353	0.272	0.023*
Age								
< 60(72)	4.54 ± 0.81	1.71 ± 1.72	1.11 ± 0.32	2.59 ± 0.73	1.26 ± 0.36	0.90 ± 0.28	217.68 ± 252.73	23.08 ± 3.40
≥ 60(115)	4.48 ± 1.02	1.31 ± 0.94	1.24 ± 0.31	2.53 ± 0.82	1.29 ± 0.25	0.87 ± 0.29	193.40 ± 267.66	23.14 ± 3.27
*P*	0.694	0.073	0.006*	0.607	0.519	0.410	0.538	0.903
TNM stage								
I(33)	4.62 ± 1.06	1.63 ± 2.22	1.26 ± 0.37	2.58 ± 0.85	1.25 ± 0.31	0.86 ± 0.29	140.42 ± 123.13	23.13 ± 3.88
II(50)	4.48 ± 0.80	1.40 ± 0.89	1.21 ± 0.31	2.50 ± 0.69	1.32 ± 0.35	0.87 ± 0.29	231.84 ± 318.15	23.46 ± 3.25
III(43)	4.50 ± 0.79	1.61 ± 1.39	1.20 ± 0.30	2.53 ± 0.71	1.33 ± 0.27	0.89 ± 0.27	200.93 ± 202.01	23.10 ± 3.16
IV(61)	4.48 ± 1.08	1.31 ± 0.76	1.13 ± 0.32	2.60 ± 0.88	1.21 ± 0.25	0.89 ± 0.30	213.90 ± 299.77	22.86 ± 3.20
*P*	0.905	0.573	0.267	0.895	0.141	0.933	0.460	0.827
N classification								
(-) (119)	4.51 ± 0.87	1.47 ± 1.37	1.22 ± 0.32	2.52 ± 0.72	1.30 ± 0.33	0.88 ± 0.28	190.40 ± 240.41	23.21 ± 3.36
(+) (68)	4.50 ± 1.06	1.45 ± 1.19	1.14 ± 0.33	2.61 ± 0.88	1.24 ± 0.23	0.88 ± 0.30	224.35 ± 295.71	22.96 ± 3.25
*P*	0.950	0.906	0.095	0.464	0.222	0.998	0.395	0.627
Location								
Tongue (87)	4.62 ± 1.05	1.40 ± 0.88	1.21 ± 0.34	2.60 ± 0.89	1.32 ± 0.33	0.91 ± 0.30	196.28 ± 275.99	22.99 ± 3.12
Buccal (42)	4.43 ± 0.85	1.48 ± 1.38	1.17 ± 0.25	2.54 ± 0.66	1.24 ± 0.24	0.87 ± 0.31	258.50 ± 334.63	23.77 ± 3.12
Lip (7)	3.89 ± 0.60	1.44 ± 0.73	1.01 ± 0.23	2.20 ± 0.63	1.11 ± 0.24	0.76 ± 0.22	98.43 ± 98.30	22.26 ± 3.57
Palate (10)	4.42 ± 0.71	2.72 ± 3.89	1.24 ± 0.55	2.28 ± 0.65	1.29 ± 0.47	0.77 ± 0.24	258.00 ± 193.10	22.98 ± 3.93
Gingiva (34)	4.60 ± 0.80	1.24 ± 0.66	1.24 ± 0.30	2.68 ± 0.71	1.28 ± 0.20	0.89 ± 0.25	148.12 ± 130.82	23.13 ± 3.92
Mandible (7)	3.78 ± 0.76	1.36 ± 0.56	0.95 ± 0.21	2.15 ± 0.53	1.08 ± 0.18	0.79 ± 0.21	239.29 ± 214.67	21.90 ± 2.80
*P*	0.100	0.058	0.203	0.326	0.175	0.508	0.402	0.679

^*^P < 0.05

### Comparison of serum lipids and BMI according to different clinicopathological parameters of OPMD

As shown in Table 4, female patients had significantly higher TC, HDL-C, and Apo-A levels than male patients among the patients with OPMD (*P* < 0.05). Patients older than 60 years with OPMD had higher HDL-C levels than younger patients (*P* < 0.05), whereas the levels of LDL-C were paradoxically higher in younger patients (*P* < 0.05). Furthermore, a total of OPMD patients with two diseases, OLP and OLK with dysplasia, were enrolled in this study. A comparison of the values in patients with OLP and OLK with dysplasia revealed that the HDL-C and BMI values in the OLK with dysplasia patients were significantly higher than those in the OLP group, and the expression levels of LDL-C, and Apo-A were lower than those in the OLP group (*P* < 0.05). In this study, we further analysed the relationships between different clinical types of OLP patients and serum lipid profiles and BMI, and found that there was no obviously high or low expression in patients with plaque type and erosion type.

**Table 4:** Comparison of serum lipid components and BMI between different subgroups in patients with OPMDs

Parameter(n)	TC (mmol/L)	TG (mmol/L)	HDL-C (mmol/L)	LDL-C (mmol/L)	Apo-A (g/L)	Apo-B (g/L)	Lpa (mg/L)	BMI (kg/m^2^)
Gender								
Male (42)	4.43 ± 0.94	1.37 ± 0.77	1.33 ± 0.53	2.31 ± 0.82	1.39 ± 0.023	1.00 ± 0.39	155.43 ± 204.57	23.83 ± 3.73
Female (103)	4.78 ± 0.82	1.31 ± 0.63	1.55 ± 0.61	2.42 ± 0.72	1.54 ± 0.20	0.96 ± 0.37	179.18 ± 260.23	23.80 ± 3.22
*P*	0.024*	0.628	0.036*	0.413	< 0.001*	0.517	0.598	0.966
Age								
< 60(101)	4.63 ± 0.85	1.32 ± 0.67	1.36 ± 0.41	2.48 ± 0.69	1.51 ± 0.21	0.96 ± 0.36	164.05 ± 229.74	23.53 ± 3.49
≥ 60(44)	4.80 ± 0.90	1.35 ± 0.67	1.79 ± 0.81	2.17 ± 0.84	1.45 ± 0.24	0.99 ± 0.41	191.25 ± 278.68	24.44 ± 2.99
*P*	0.282	0.807	0.001*	0.039*	0.149	0.662	0.541	0.135
Disease								
OLP (105)	4.70 ± 0.92	1.28 ± 0.63	1.33 ± 0.29	2.57 ± 0.70	1.53 ± 0.21	0.96 ± 0.38	173.73 ± 260.80	23.45 ± 3.42
OLK (40)	4.64 ± 0.71	1.43 ± 0.75	1.90 ± 0.91	1.91 ± 0.66	1.40 ± 0.22	1.00 ± 0.38	168.55 ± 200.38	24.75 ± 3.02
*P*	0.725	0.241	< 0.001*	< 0.001*	0.001*	0.599	0.910	0.037*
Type (OLP)								
Reticular (45)	4.72 ± 1.01	1.30 ± 0.69	1.30 ± 0.28	2.64 ± 0.75	1.51 ± 020	1.00 ± 0.33	153.67 ± 184.01	22.83 ± 3.58
Erosive (60)	4.68 ± 0.86	1.27 ± 0.59	1.35 ± 0.30	2.51 ± 0.66	1.55 ± 0.21	0.93 ± 0.41	188.78 ± 306.76	23.91 ± 3.26
*P*	0.826	0.804	0.350	0.351	0.268	0.330	0.497	0.110

^*^P < 0.05

### Regression analysis of lipid levels and BMI values of OSCC and OPMD

**Fig. 1:**
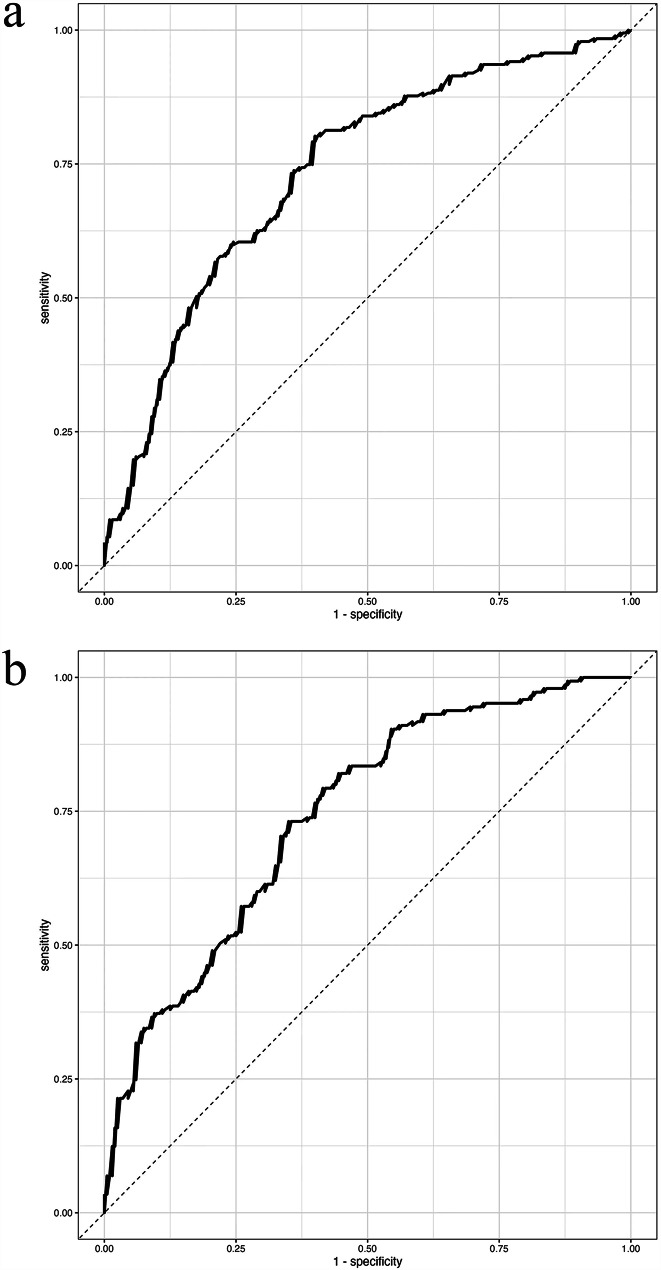
Regression analysis regarding the occurrence of OSCC and OPMD. (a) ROC curves of regression models for the OSCC group: the AUC value was 0.734. (b) ROC curves of regression models for the OPMD group: the AUC value was 0.742

Whether the patients had OSCC or OPMD was the dependent variable (0 = benign lesions, 1 = OSCC or OPMD). The serum lipid levels and BMI values were categorized into normal, low and high groups according to the laboratory reference range and used as categorical variables. Age was also included as a continuous variable in the logistic regression analysis. The results showed that age and lower levels of Apo-A were associated with a higher risk of developing OSCC (Table 5). The receiver operating characteristic (ROC) curve revealed that the area under the curve (AUC) value was 0.734, which indicated that the model performed well (Fig. 1a).

**Table 5:** Logistic regression analysis of developing OSCC

Predictors	Odds Ratios	95%CI	*P*
Gender			
male	1		
female	0.98	0.62–1.55	0.924
Age	1.07	1.05–1.09	< 0.001*
BMI (kg/m2)			
18.5–23.9	1		
< 18.5	1.75	0.66–4.77	0.261
> 23.9	0.8	0.50–1.27	0.345
TC (mmol/L)			
0-5.2	1		
> 5.2	1.62	0.66–4.08	0.293
TG (mmol/L)			
0.4–1.81	1		
< 0.4	0.35	0.01–3.79	0.433
> 1.81	1.53	0.85–2.79	0.16
HDL-C (mmol/L)			
1.07–1.9	1		
< 1.07	1.01	0.60–1.70	0.96
> 1.9	1.07	0.26–4.59	0.922
LDL-C (mmol/L)			
0-3.1	1		
> 3.1	0.88	0.37–2.05	0.761
Apo-A (g/L)			
1-1.6	1		
< 1	2.33	1.04–5.44	**0.043** ^*^
> 1.6	0.78	0.34–1.75	0.548
Apo-B (g/L)			
0.1–1.81	1		
> 1.81	0.76	0.19–2.92	0.696
Lpa (mg/L)			
0-300	1		
> 300	0.87	0.50–1.51	0.627

^*^P < 0.05

This study also found that sex, HDL-C and Apo-A were risk factors for OPMD. Females were more likely to develop OPMD, and higher HDL-C and Apo-A values were associated with a greater probability of developing OPMD (Table 6; Fig. 1b).

**Table 6:** Logistic regression analysis of developing OPMD *P<0.05

Predictors	Odds Ratios	95%CI	*P*
Gender			
male	1		
female	2.54	1.55–4.21	**< 0.001** ^*^
Age	0.98	0.96–1.00	0.064
BMI (kg/m2)			
18.5–23.9	1		
< 18.5	0.3	0.08–1.01	0.06
> 23.9	0.98	0.59–1.63	0.941
TC (mmol/L)			
0-5.2	1		
> 5.2	2.89	0.99–9.85	0.066
TG (mmol/L)			
0.4–1.81	1		
< 0.4	0.21	0.01–1.83	0.199
> 1.81	1.27	0.66–2.45	0.469
HDL-C (mmol/L)			
1.07–1.9	1		
< 1.07	0.68	0.36–1.26	0.224
> 1.9	5.01	1.57–19.09	**0.01** ^*^
LDL-C (mmol/L)			
0-3.1	1		
> 3.1	0.35	0.10–1.05	0.077
Apo-A (g/L)			
1-1.6	1		
< 1	0.54	0.10–2.09	0.409
> 1.6	2.25	1.16–4.46	**0.018** ^*^
Apo-B (g/L)			
0.1–1.81	1		
> 1.81	2.05	0.58–7.39	0.262
Lpa (mg/L)			
0-300	1		
> 300	0.82	0.44–1.51	0.53

## Discussion

OPMD are at a high risk for transformation to OSCC, and lipid metabolism disturbance is a common occurrence in patients with tumours [13]. Tumours are known to occur with dyslipidaemia. The expression of serum lipids might be associated with their phenotype, and the low levels of HDL-C and LDL-C were related to oestrogen receptor-negative and progesterone receptor-negative breast cancer. Well-controlled serum lipids might regulate the tumour immune microenvironment and prognosis of postmenopausal hormone receptor positive/human epidermal growth factor receptor 2-negative breast cancer patients [25, 26]. High TGs and TC levels increased the risk of developing colorectal cancer, whereas high HDL-C levels were inversely associated with colorectal cancer incidence [27]. Dyslipidaemia also increased the risk of developing non-small cell lung cancer [15, 28]. Lipid abnormalities also contributed to liver carcinogenesis. Mice with high cholesterol levels after the injection of certain chemical carcinogens or hepatoma cells developed smaller tumours [14, 29–31]. According to the researchers, TGs and TC might serve as potential predictors of overall survival in cervical cancer [17]. Dyslipidaemia is also evident in some metabolic diseases, such as diabetes mellitus, polycystic ovary syndrome, and cardiovascular disease [32–34]. Moreover, the previous studies on the expression differences of each lipid profile in HNSCC have reported inconsistent findings [21, 22, 35], which prompted us to further investigate the lipid profile in OSCC and OPMD patients to provide new ideas for clinical diagnosis and treatment.

This study found no significant differences in the levels of TC, TGs, HDL-C, LDL-C, Apo-A, Apo-B, Lpa and BMI values between the control individuals and the 187 OSCC patients, which was inconsistent with previous studies in HNSCC patients [21, 22]. One reason for this may be that Li et al. performed paired studies, while this study was not able to adjust for differences in diet, lifestyle, etc. However, this study did adjust for differences in sex and age. Another reason might be that the subjects of Li et al. were patients in 2009–2014, which was almost 10 years prior to our study. During this time, changes in social factors, patients’ diet, lifestyle habits, etc., might have contributed to the inconsistent results. However, interestingly, the levels of HDL-C, Apo-A and Apo-B were lower in the OSCC group than in the OPMD group, which suggests that dyslipidaemia changes during the carcinogenesis of OSCC. Moreover, clinicopathological data of OSCC patients were analysed in this study, finding that Apo-A and BMI were differentially expressed between male and female patients. Apo-A, the major structural protein of HDL-C, can activate the process of free cholesterol esterification, and transport excess cholesterol from tissues to the liver. BMI is a measure of overall adiposity. In the present study, the levels of Apo-A and BMI values were higher in females than in males. This finding indicated that females with obesity and elevated Apo-A levels may be more likely to develop OSCC, or there might be implications related to differences in region, diet, and so on. Consistent with OPMD, higher values of HDL-C were observed in OSCC patients who were over 60 years of age, implying that the cholesterol of older patients was more likely to be transported to the liver. Further study indicated that lipid levels were not correlated with TNM stage, lymph node metastasis, or tumour location in OSCC patients, which was consistent with previous findings [21]. These results may indicate that altered lipid levels are independent of cancer type. Regression analysis indicated that age was a risk factor for OSCC occurrence, which was consistent with the results of previous studies [36, 37]. However, the results did not show the effect of sex on the disease, which may be related to the insufficient sample size.

This study also explored the relationship between OPMD and blood lipids, and found that, unlike OSCC patients, patients with OPMD had higher HDL-C and Apo-A values than control individuals, which was consistent with the following regression analysis. The main role of HDL-C is to reverse cholesterol transport, and cholesterol plays an important role in safeguarding tissue metabolism [38, 39]. In the Table 2 of this paper, it was found that although there was no statistical difference in the expression of TC between the OPMD and the normal group, the TC level of OPMD patients was generally on the rise. Previous literature indicated that after depositing cholesterol, the HDL-C was re-secreted to transport cholesterol [18, 40]. Thus, we speculated that changes in TC levels lead to an increase in HDL-C and Apo-A in OPMD patients. Our subgroup analyses showed that female patients with OPMD had higher lipid levels than male patients. Female sex, and higher levels of HDL-C and Apo-A were risk factors for the development of OPMD, suggesting that we should be clinically alert to the risk of OPMD in dyslipidaemic female patients.

Finally, logistic analysis indicated that low Apo-A levels were associated with the occurrence of OSCC, which was consistent with previous studies in lung, liver, colorectal, breast, prostate and hematologic malignancies [41, 42]. Interestingly, logistic analysis also showed that high HDL-C and Apo-A were related to the occurrence of OPMD, which was not reported in the previous literature. According to the above analysis, dyslipidaemia was found in patients with OPMD, while the serum lipid levels returned to normal levels after transformation of OPMD into OSCC. In OSCC patients, the lipid levels were basically consistent with those of control individuals.

This study comprehensively analysed the continuous changes in blood lipids during disease progression. However, information on alcohol consumption and smoking was not collected in this study. Obesity was assessed by BMI in this research, but BMI is a composite measure that cannot represent body fat distribution. Information on patients from Nanjing and the surrounding area cannot accurately reflect the overall level of the whole society due to the differences in diet and region. The mechanism underlying the associations between blood lipids and diseases was not explored. Limitations of the current study also include the lack of follow-up.

## Conclusion

In summary, serum lipids play a role in the occurrence and development of OSCC. Patients with OPMD might have abnormal lipid levels, and the occurrence of OPMD might be related to sex and the higher levels of HDL-C and Apo-A. No significant lipid-abnormalities were observed in the patients with OSCC, while age and a lower Apo-A value might be risk factors for developing OSCC.

## Data Availability

All data generated or analyzed during this study are included in this article.

## Abbreviations

OPMD: Oral potentially malignant disorders
OSCC: Oral squamous cell carcinoma
TC: Total cholesterol
TGs: Triglycerides
HDL-C: High-density lipoprotein
LDL-C: Low-density lipoprotein
Apo-A: Apolipoprotein A
Apo-B: Apolipoprotein B
Lpa: Lipoprotein (a)
OLK: Oral leukoplakia
OLP: Oral lichen planus
PVL: Proliferative verrucous leukoplakia
OSF: Oral submucous fibrosis
HNSCC: Head and neck squamous cell carcinoma
BMI: Body mass index
TNM: Tumour-node-metastasis
ROC: Receiver operating characteristic
AUC: Area under the curve.
